# “A woman’s honor tumbles down on all of us in the family, but a man’s honor is only his”: young women’s experiences of patriarchal chastity norms

**DOI:** 10.1080/17482631.2020.1862480

**Published:** 2020-12-20

**Authors:** Monica Christianson, Åsa Teiler, Carola Eriksson

**Affiliations:** Department of Nursing, Umeå University, Umeå, Sweden

**Keywords:** Gender, grounded theory study, honour, women’s health, violence against women

## Abstract

**Purpose**: In this qualitative study we explored how young women living in Sweden with ethnic and cultural roots in the Middle East and East Africa comply with or resist so-called honour norms and how they perceive that these norms affect their living conditions.

**Method**: In depth interviews were performed with 14 young women. The majority were between 21 and 32 years of age with a mean age of 24. All interviews were transcribed verbatim and a grounded theory approach was used. To reflect the diversity in women’s experiences, the grounded theory approach was conducted from a feminist perspective to transform women’s personal narratives to a larger social context.

**Results**: We analysed the core category “Honorable women in becoming” as the central emerging phenomenon related to categories about structural and individual control of women, the women’s adjustment and resistance, and the continuum of severe consequences and violence that they experienced in their struggle for autonomy.

**Conclusion**: Simone de Beauvoir’s feminist theory about women as “the other” was an inspiration and gave us valuable input to highlight women’s experiences and situations from a perspective of gender, power, and oppression.

## Introduction

In many collective patrilineal societies, the norms and ideals surrounding women’s appearances, behaviours, and life choices are strongly associated with the entire family’s morals and honour (Cihangir, 2012; Cooney, 2014; King, 2008; Leung & Cohen, 2011). In the Middle East, gossip, scandal, and shame are cultural dimensions aimed at controlling and dominating women, particularly daughters and wives (Awwad, 2001). Surveys from Egypt, Palestine, Israel, and Tunisia indicated that the concept of family honour is centred on the notion that it is especially women’s responsibility, to behave in a way that protects the honour of the family (Douki et al., 2003; Rodriguez Mosquera, 2013). Interviews with young women from racially/ethnically minoritized communities in Sweden indicate that negative reputation brings shame both to the young women and their families (Cinthio, 2015). In this empirical interview study, we have examined the existence and experience of honour norms among young women living in Sweden from ethnically and culturally diverse backgrounds.

Honour codes (either as social practices or ideological constructs) prescribe how both men and women should maintain the social reputation of their family; however, the premises are dramatically different for men and women (Fildis, 2013). According to King (2008), being able to protect the well-being of the family, being virile, and upholding authority within the family are core attributes associated with the “masculine” honour code. The “masculine” honour code can be considered to benefit men because it leaves room for men to behave autonomously having the freedom to do what they want (Cihangir, 2012), and, at the same time, take power away from women.

The “feminine” honour code emphasizes values such as chastity and conformity. The central ideal in the “feminine” honour code is sexual purity, modesty, decorum in dress, and discretion in social relations, particularly with men, or inhibit choice of marriage partner (Sen, 2005). This code is an important indicator of the status of the family’s honour, so protection of the family on the part of men extends beyond physical and economic protection as control of a female’s sexual behaviour is also the responsibility of the male family members (Cihangir, 2012). Not being able to live up to such responsibility translates into a perceived loss of masculinity for male relatives and this may result in very severe punishments for women (Cihangir, 2012; Fildis, 2013). Consequently, young women can be controlled both at home and socially where male dominance as well as female subordination is standard (Schlytter & Linell, 2010). However, some family members may oppose these norms, which can be very risky. Family honour is threatened if a female family member is perceived to violate the “feminine” honour code (Schlytter & Linell, 2010).

Restoring the honour of the family often results in extreme corrections, so-called honour violence, that can include social isolation, psychological and physical mistreatment, domestic violence, forced suicide, forced marriage, marital rape, and even murder (i.e., honour killings) (Awwad, 2001; Cooney, 2014; King, 2008; Sen, 2005; Simga, 2019). The UN states that honour violence represents a form of systematic institutionalized misogyny that includes the full spectrum of discrimination and violence committed against women involving power, control, domination, and intimidation to preserve patriarchy (United Nations, 2014).

Honour violence is described as actions that are performed within the family where it is common for these actions to be sanctioned collectively and in some cases to be socially as well as legally acceptable (Awwad, 2001). Sen (2005, p. 50) argues that there are some key features that characterize honour violence: gender relations that problematize and control women’s (sexual) behaviours, the role of women in monitoring other women’s behaviours, collective decisions regarding punishment, the potential for women’s participation in killings, the ability to reclaim honour through enforced compliance or killings, and state sanction of honour killings.

Honour violence exists all over the world (Kulczycki & Windle, 2011; Sen, 2005). It is a global problem, and the arrays of the violence can be regionally distinct, but the causes emanate from gender-based and socio-economic inequalities (Bhanbhro et al., 2016). Various studies have found that women may “dishonor” their family by being perceived to have violated female chastity norms, including loss of virginity before marriage, infidelity after marriage (Baker et al., 1999; Cooney, 2014; Faqir, 2001) or interacting socially with men outside the family (Akpinar, 2003), and acting autonomously by, for example, gaining an education, securing a job, leaving an abusive husband or dressing how she wants (Hasan, 2002; Ince et al., 2009).

In 2000, United Nations Population Fund (UNFPA) estimated that 5000 women were victims of honour killings each year (United Nations Population Fund, 2000), but as honour based violence is wider than honour killing, this is a silent and hidden problem that mostly hits women, and the real numbers may be much higher (Awwad, 2001). According to statistics from the Honour Based Violence Awareness Network (http://hbv-awareness.com/statistics-data/) the true prevalence of honour based violence at the current time is unknown and very difficult to estimate. In the Middle East and North Africa, victims of honour killings are mostly young women murdered by a man to whom they are related, but very little is known about the incidence, correlates, and predictors of honour killings in the region, and much more research on family dynamics is required (Kulczycki & Windle, 2011).

Research on honour codes has often been focused on the Mediterranean, Middle East, and South Asian countries, but Rodriguez Mosquera et al. (2002) found in their cross-cultural study of Spanish and Dutch youth that Mediterranean conceptualization of honour is also present in Northern Europe even if the expressions are less visible. In Western Europe today, there has been a transformation of motives where to live one’s life and to make one’s own life choices can be the motive (Grzyb, 2016).

In Sweden, the extent of so-called honour violence is largely unknown, although research is pointing to a widespread problem where women who are experiencing honour violence are at higher risk for both physical as well as mental health problems (The National Centre for Knowledge on Men’s Violence Against Women, 2011). The policy of honour based violence in Sweden reflects the UN definition and academic frameworks on honour and gender norms, where honour based violence can be described as norms built on strong patriarchal and heteronormative perceptions, grounded in gender, power, sexuality, and culture (SoU, 2018). From this view (SoU, 2018), the interests of the individual are subordinated to the interests of the family, relatives, or clans. The control of girls and women goes from “everyday forms of limitations” such as clothing, social relations, freedom of movement, life choices, education, work, marriage and divorce to female genital mutilation, forced marriage, child marriage, threats of violence, and lethal violence (SoU, 2018).

While data is limited, it is estimated that 70,000 youth experience limitations in their choice of partner, around 8500 women are worried about being forced by their family to marry against their will, and about five women are murdered because of honour every year (Swedish Agency for Youth and Civil Society, http://gapf.se/rapporter-och-statistik/), Young men’s dual role as potential perpetrators and/or victims has also received some attention (Rexvid & Schlytter, 2012), but in general, it is men who commit honour violence against women when women seek to make independent lifestyle decisions (Cooney, 2014; Kvinnoforum, 2003).

Although the international and nation media, human rights groups, and politicians have raised awareness about honour violence, the main contribution to awareness about honour violence has been raised by honour crime victims in the last decade. There is still little knowledge about how young women, from communities where honour codes are significant, experience upbringing, living conditions, and equality. To this end, this study explored how young women living in Sweden with ethnic and cultural roots in Middle East and East Africa comply with or resist patriarchal chastity norms and control and how they perceive that these norms affect their lives.

## Material and method

A qualitative methodology was chosen as this approach is exploratory and captures the multiple realities, views and emotions of the phenomenon under study (Lincoln & Guba, 1985). To perform a critical inquiry and to produce a thoughtful analysis, constructive grounded theory was suitable (Charmaz, 2017). Therefore we conducted in depth interviews with 14 women with various experiences of honour, in the capital of Sweden and in one university town in the north part of Sweden

### Sample and analysis

A purposive sampling strategy was performed. To be eligible for the study, the informants had to be young women over 18 years of age living in Sweden from communities where honour codes are significant. Recruitment occurred between 2012 and 2015.

Women were asked to participate in the study through key persons at youth clinics (i.e., director of department and midwives), women’s organizations working against violence against women (directors of organizations), and healthcare centres (floor manager). Snowball sampling generated one informant. Contact information to potential informants were provided.

The sample consisted of 14 informants who lived in different cities in Sweden. Eleven informants originated from countries in the Middle East and East Africa, while three informants were born in Sweden. Their parents were from Egypt, Kurdistan, Lebanon, Iran, Iraq, Palestine, Turkey, and Somalia. Eight informants migrated with their parents to Sweden in their early childhood and have been studying in Swedish schools. Two informants migrated to Sweden when they were adults and one informant came to Sweden as a refugee when she was a teenager. The majority were between 21 and 32 years of age with a mean age of 24. Their living situation varied from sheltered accommodations to living on their own or with a boyfriend, husband, or their parents (Table I).

The women received both written and verbal information about the study. The interviews were performed by the researchers responsible for the project, both skilled in interview techniques and grounded theory analysis.

**Table I.** Group-level demographic background data; in-depth interview study with 14 women

Age	Ethnic background/ ancestral homeland	Education	Civil status	Number of children	Form of housing
19–38 Mean age 24	Egypt (1) Iran (2), Iraq (3), Lebanon (2), Turkey (1), Somalia (2), Sweden (3)	Senior high school (2) University student (6) University exam (6)	Single (7) Cohabiting (2) Married (2) Divorced (3)	0 (11) 1 (1) 2 (2)	Single housing (7) Cohabitation (3) Living with parents (2) Women’s Shelter (2)

Before the interview started, the informants were given the opportunity to ask questions about the project. All interviews were carried out in a respectful manner where the women were encouraged to share their own opinions, feelings, and thoughts in a way that we perceived did not create distance between interviewees and the interviewer. Before the interview started, the informants completed a questionnaire that asked them to provide basic socio-demographic information.

The in-depth interviews lasted between 60–90 minutes and took place at a location that the interviewees chose. In one case, the interview was conducted via telephone. An interview guide was developed with open questions about the interviewee’s upbringing, living conditions, and values about equality, honour, virginity, hymen reconstructions, sexuality, and relationships. This article focuses what women said on upbringing, living conditions, and equality. All the informants agreed to have the interviews recorded. Clarifying and follow-up questions were asked during the interviews to obtain a rich, detailed narrative. We ended the interview by asking the informants if they had more information to share with us. All interviews were transcribed verbatim. In order to ensure anonymity, pseudonyms are used here for the informants.

The Regional Ethical Review Board at Umeõ. University, Sweden approved the study in 2011 (dnr 269–31 Ö). The study was conducted according to general ethical guidelines in the Helsinki Declaration (World Medical Association, 2013). Prior to participation, information about the study was given to the young women in writing, and oral and written informed consent was obtained from all informants. They were informed that their participation in the study was voluntary, and that they had the freedom to withdraw their participation at any time without explanation.Steps to preserve confidentiality and privacy were taken, including removing names and other identifying information. Only participants aged 18 and older were interviewed. Any information that might allow identification has been removed.

### Analysis

We used a grounded theory approach. Grounded theory is an inductive method that discovers, creates, and verifies the phenomenon it studies through systematic data collection and analysis (Strauss and Corbin, 1990). The inductive nature of the method assumes openness and flexibility. Thus, you follow the leads driven by the data, not from the literature review of the traditional research design. A fundamental postulation of grounded theory is to let the key issues emerge instead of forcing them into preconceived categories (Charmaz, 2006). We focused on what our research participants talked about and how they explained their statements and actions. As such, we ask what analytical sense we can make of them to construct theories “grounded” in the data. To reflect the diversity in women’s experiences, a grounded theory method can be conducted from a feminist perspective to transform women’s personal narratives to a larger social context (Wuest, 1995). There are a shared set of common epistemological values guiding a feminist perspective that promotes compassion and understanding. For example, women’s lived experiences are valued as legitimate sources of knowledge, knowledge is generated through social processes, subject-object dualism is rejected, and all research encompasses subjectivity, partiality, and bias and can promote social change (Plummer & Young, 2010).

The combination of grounded theory and feminist perspectives helps protect against androcentric views and has great potential to reveal issues particular to the experiences of marginalized women (Plummer & Young, 2010) This approach was useful, as, in line with Charmaz (2006, p. 130), we understand grounded theory as a constructivist approach that prioritizes the phenomenon of study where both data and analysis were created in interaction with the informants and other data sources.

The first and second author analysed and interpreted the data in collaboration with the third author, according to the three steps in grounded theory as described by Strauss and Corbin (1994): open coding, axial coding, and selective coding. In the initial phase, the open coding, we scrutinized the data several times and read through the interviews to conceptualize the data by asking questions such as “What is this?” and “What does it represent?” Codes were assigned and compared for their properties and dimensions to capture the actions, feelings, and viewpoints described by the informants and to maintain closeness to the text. A preliminary analysis was carried out after the first four interviews where we discovered a provisional pattern of subordination and control. Thereafter we performed ten more interviews to deepen what was relevant to the emerging area under study and in reciprocity with the gender analysis. The second phase, axial coding, went hand-in-hand with open coding. In axial coding, we developed sub-categories that were eventually divided and linked to other categories. In this phase, we further developed the range of properties and dimensions and compared and sorted the codes. This phase occurred continuously during discussions in the research group until consensus was reached. As a result, our analysis constitutes one reading of the data rather than the only truth about the data.

We produced memos throughout the process as these are important pieces in the analysis. Memos, informal analytical notes (Charmaz, 2006, p. 87), accelerated the productivity during the analytical process. In the final analysis, we checked the memos and compared them to the generated text and asked questions such as “Does this bring anything new to the analysis?” The memos increased our confidence to develop fresh ideas and create new concepts that strengthened the findings.

The use of primary sources of data, i.e., the inclusion of quotations from all participants added to credibility (Strauss and Corbin, 1990).

The preliminary “honorable women” concept emerged during the process of selective coding and at this point we covered the literature about Simone de Beauvoir’s theory about women’s situations and women as “others”, as described in *The Second Sex* (1949/2012), which was woven into our work explicitly. In this phase, the core concept emerged that was linked to all categories (Charmaz, 2006), and the core concept was inspired by Simone De Beauvoir’s (1949/2012) elaborations about femininity.

### Theoretical framework

de Beauvoir’s autobiographical essay is commonly described as launching the contemporary feminist movement. She elaborates on the dimensions that define “woman” and the central question concerning the identity of “woman” (Hekman, 2015). de Beauvoir argued that women had fundamental rights to work, to contraceptives, to independence, and to take part in society as much as men (Lundgren- Gothlin, 1991, p. 353). She defended the human rights for women in a period when women were denied these rights, so important then and so important in contemporary Sweden, where women and men should have equal power to shape the society and their own lives (Gender equality in Sweden). de Beauvoir’s theory is relevant in contemporary society, especially her central emphasis on the situation and oppression of women. Likewise, Hekman interprets and addresses de Beauvoir’s feminist concept as impossible to separate women from their situation in the society: “this situation defines everything: her identity, the possibility of freedom, the possibility of change” (2015, p. 148). From that point of departure, Simone de Beauvoir’s essays about femininity gave us valuable input to highlight these women’s experiences from a perspective of gender and power. In her book *The Second Sex* (1949/2012), Beauvoir formulates the important question “what is a woman?” and suggests that women are differentiated in relation to men. Toril Moi (2011, pp. 4–5) claims that “no feminist has produced a better theory of the embodied, sexually different human being than Simone de Beauvoir in *The Second Sex*”, as she provides a non-essentialist, solid historical and social understanding of women, with resonance in contemporary societies.

Simone de Beauvoir argues that men are seen as subjects and women are seen as objects; men represent the norm and women represent “the other”. Throughout history, women have been treated as inferior to men, a view that she argues is not based in biology or nature but a creation of society, a socially-constructed concept. From de Beauvoir’s perspective, the world belongs to men and they offer women protection and justifications for her existence. According to Beauvoir, women’s invisibility throughout history is a result of living in a patriarchy. Women want to free themselves from paternalism but are “kept in place” by men. They are seen as inferior and subordinated to men, leaving them with fewer possibilities. According to Beauvoir, femininity is a socially-constructed category that is denying women autonomy, authority and places women’s identities in relation to men: “One is not born, but rather becomes a woman” (De Beauvoir, 1949/2012, p. 267). That is, a woman’s destiny is imposed on her by society. She might have been born female, but it is society that creates a woman, decides what is feminine, and dictates how women should behave. For Beauvoir, feminists fight for the right for women to be just as important and relevant as men, a concern that includes more than just political and economic equality. According to Moi (2011, p. 83), what Beauvoir did in her book *The Second Sex* was to call attention to a devastating critique of sexism, where women can be not only the victims of sexism but also the potential antagonists to oppressive social norms. From that point of view, Beauvoir’s theory is a useful source and inspiration and thus relevant for our study where young women’s voices are heard.

## Findings

**Figure 1.**
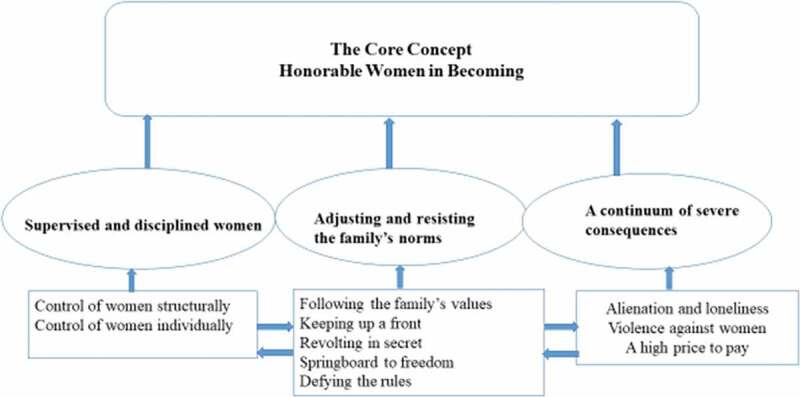
A flow chart of how the web of collective and individual actions shape women

The core category “Honorable women in becoming” was analysed as the central emerging phenomenon to which all the categories are related throughout the findings. Simone de Beauvoir’s quotation “One is not born, but rather becomes a woman” (De Beauvoir, 1949/2012, p. 325) reflects how most of the women were shaped into the honourable woman role. The first category—“Supervised and disciplined women”—describes how women are perceived to be controlled both structurally and individually. The second category—“Adjusting and resisting the family norms”—shows how women handled their potential subjection. The third category—“A continuum of severe consequences”—addresses how their lives are affected in their struggle for autonomy. Figure 1 is a flow chart of how “ideal” women are constructed.

The flow chart (Figure 1) offers an imaginative interpretation concerning how the various subcategories, categories and the core concept are interrelated both individually and collectively in a bottom-up design. The subcategories are interpretations of the narratives that are drawn from the individual micro level actions, whereas the categories constitute the causes and consequences that we interpret as the structural level of actions that lead to the “top” level core concept of the “ideal” feminine gender construction——in line with being honourable.

### Supervised and disciplined women

#### Control of women structurally

The women that we interviewed argued that social oppression against women was supported by a strong cultural heritage re-inscribed by patriarchal clan systems, traditions sustained by governments, and society as a whole. These norms exist in the countries of their parents’ origin and were supported by institutions on different levels. According to Ciwan, the state, for example, in Iran and Iraq often reinforced the control over women with unequal legislation for women compared to men where “honor-related crimes” were not seen as criminal offences:

Yes, everyone talks about it, within Islam, a girl who has a sexual relationship without being married is the greatest shame for a family and she will never be forgiven, and morally she is seen as a sinner and therefore must die. Even the government thinks so.

Social influences were perceived to normalize honour values by propounding stereotypical views of women as weak, uneducated, and dependent on men. Some women claimed that men viewed them as objects and merchandise that could be traded among men. A key aspect of this objectification was maintaining a woman’s reputation. A respectable woman dresses properly, keeps a flawless façade, and does not expose body parts perceived as feminine. From some women’s point of views, women were not perceived to be autonomous subjects with their own free will. They were forbidden to talk to men in public and it was recommended that they stay at home or were not allowed to go out alone to “protect” women from situations where women’s reputation could be damaged. These oppressive actions were often connected to denying women similar human rights as men, such as the right to exercise and to have sexual and non-sexual relations with men. The honour concept imprinted the views on women as the collective carriers of maintaining men’s honour by behaving in line with the “feminine” honour code, which from Alicia’s point of view, was perceived as a double burden for women: “A woman’s honor tumbles down on all of us in the family; a man’s honor is only his”.

#### Control of women individually

According to many of the women, their parents were described as limiting their life space by restricting and disciplining them to follow the patriarchal norms. Furthermore, the parents controlled and disapproved of their companions and their activities. Some women claimed that their parents struggled to uphold old-fashioned traditions, where often both the mother and the father were involved in the control and settings of limits, making the women feel powerless, and not autonomous subjects with their own free will. Sara said:

And before it gets dark, I have to come home and they have checked how much time it takes back and forth. For example, if I’m five minutes late or something, there will be a quarrel, and they say ‘where have you been? What did you do?” [… .] they are afraid that I meet boys, because, according to them, that is not a normal thing to do.

Many women claimed that as a marriage strategy they were forced to focus on their reputation, to behave nicely, and to not cast shame on the family. Jian revealed:

I have to be home earlier and I have always had restrictions on which I could and could not hang out with. It is very important to socialize with good people, to not get a bad reputation. That reputation might then damage me later, because then someone might not want to marry me.

Fathers were often portrayed as the head of the household, exercising their verbal or physical power if their daughters disappointed them or misbehaved. The women preserved a façade to protect the family honour. However, the fathers were often described as largely absent during the women’s childhood because of migration, work abroad, divorce, or being dead. Some of the women highlighted that their brothers inherited the role of the father by controlling their sisters by “imprisoning” them or setting ultimatums when disliking a boyfriend. The person controlling them could change; for example, after marriage, the husband took over the paternalistic authority. The mothers were perceived to have a difficult role in supporting men’s ideas about how noble women should be formed. Mothers were described as co-actors in maintaining the traditions and raising their daughters to become honourable. They were sometimes portrayed as the dominant supporters of the patriarchal values and were seen as oppressing their daughters. Evin shared her experiences:

It is my mother. She is the most dominant; she is like the man in the family (laughs). My father is like … well he … he helps at home and so, but he has always been away, has worked so much that you never see him at home. So my mother has always been the one that tended to us the most … And she makes the rules and my father … He says if they are good or bad.

Most of the informants had a positive attitude about their mothers, and they saw their mother as a mediator in the family, meddling between the father and the daughter, and being the role model and the head of the family with positive intentions for their daughters, for example, encouraging education before marriage. In line with this, Alicia’ experiences does not support this theme of control of women individually as she claimed that her parents’ education was a key issue in terms of her own experience of how independence and trust characterized her upbringing:

I have had the luck to be raised in a home where both my parents are working, and my dad is cooking wonderful food, so for me that is the normal status, i.e., equality between men and women on equal terms. It is not normal for me with inequality.

### Adjusting and resisting the family norms

#### Following the family’s values

A deep-rooted respect for the family made women consider how to become an “ideal” woman. They understood the families’ opinions and were adjusting to their values. They often adapted to their family’s beliefs about femininity and accepted these as everyday part of life. Aisha, who was born in Somalia thought that head scarves were perceived as a “natural” piece of clothing.

I have had the headscarf since I was very young … I was like 12 or 13 perhaps and did that because my mother and my aunt always carry head scarfs and I never thought about religion … I wanted to imitate the other women … exactly like little girls do when they take on make-up to be like the older girls.

Refraining from alcohol and smoking was a way to show respect. Some of the women appreciated their upbringing, which they claimed was different, better, and more caring compared with the way Swedish children are raised. These interviewees thought the Swedish parents were less attentive to their children. When socializing with family and relatives, many women chose modest clothing, whereas Salma expressed that she had one type of dress code at home, which was more covered, and a Swedish style outside the home wearing, for example, a low-cut neckline. Wearing clothes like small tight skirts or low-necked t-shirts was seen as provocative and westernized and by being dressed properly could be viewed as a way to show respect for the family values and not compromise the man in the family:

So, we grew up thinking “about what signals [we] are sending”. Clothes have always been important. I do not wear a head scarf, but I do not dress in a low-cut neckline because I have been brought up that it does not make a good impression. So my siblings and I have always had that in the back of the head and thought about what signals one sends, especially as a girl. I respect their opinions and accept how they think.

#### Keeping up a front

Fear of reprisals was a relatively common motivator for adjusting to family rules. To be with the family, to not stay out late at evenings, and to choose friends that the family approved were described. Not having a boyfriend or male friends was seen as a way to protect oneself from punishment. Many women reconstructed their family situation when they mingled with their school mates as a way to be accepted. The women often kept a façade, withholding information about the control and limitations they experienced to protect their family from being judged by Swedish people as being “backwards”. Sometimes they shared their experiences with friends from similar backgrounds. The women played along with the family’s rules and kept a straight face, laying low so they could “choose their fights”. To be dedicated to sports, stay away from home, and avoid thinking about the family were sometimes helpful ways to escape their perceived subordination. Alexia described:

We were a couple of girls who spent a lot of time together. Every day. Hanging at the youth centre, playing hand ball. […] And I think that just being with them and being a human outside, it rescued me at home because I could, the short moments I was at home, I could, like, turn off. I was there and saw all the shit that happened and violence and everything. When I was outdoors, the situation did not exist.

#### Defying the rules

Most of the women advocated for their right to live their life as they chose. This ranged from pleadings, discussions, arguments, and conflicts with family members and sometimes this resulted in terminating all contact with their family.

The arguments usually concerned the women’s will to navigate their lives, making their own choices, and, more importantly, taking a stand and not letting their parents defy the rules. Emmy described the confrontations as a constant struggle:

It is fine now but it has not become that way without struggle. So I’ve, gradually, almost daily really, brought up these kind of things at home for it to be an issue whatsoever. To prevent it from becoming a shock when I, for example, have a relationship or choose not to marry, type, that kind of things.

Many of the women distanced themselves and limited their contact with family members, and this varied from avoiding talking with them to excluding all contact and denying their existence. One of the women perceived it as easy to terminate all contact with her father but had limited secret contact with her mother. The women often described living in two cultural contexts—Sweden and their parents’ home country. This conflict often resulted in feelings of alienation. To distance themselves from cultural norms that they perceived as negative was important. Some families required their daughters wear a veil and some women refused as an act of independence. Others questioned the rules concerning food or modest clothing, leading to conflict with the family over their “Swedish” mindset.

Some of the women distanced themselves from citizens from their culture, seeking the company of young people with Swedish ethnicity, such as intermingling with Swedish friends, or preferring Swedish values, or choosing Swedish boyfriends often lead to conflicts according to Alexia.

My mom could say, “You have a new necklace”. And I said, “Yes, I got it from my boyfriend.” I said it several times: “His name is X, he is from Sweden.” She just said, “Be quiet!” and walked away.

Some families tried to arrange marriages for their daughters. This caused an uproar among the women, who did not want to be in an arranged marriage, wanting to marry a partner they chose. Their free will concerning their choice of partner was worth fighting for. Some distanced themselves from thinking that limited their possibilities to choose a husband or boyfriend who belonged to another community or religion, often leading to pressure to end an “improper” relationship. Hani explained:

We are close clans but between these clans they are like enemies. So they are like, they do not even greet each other. And my father did not want me to marry someone from the other clan, and his brothers too did not want that. And they were like. […] You will not marry this guy that you bring with you now. You must leave him.

#### Revolting in secret

Withholding the truth and misrepresenting events were ways for women to protect themselves from reprisals of the family. For example, Jasmine would erase all text messages, lie about relationships, or use false alibis to cover up actions the family would not approve because she feared the consequences:

I have been lying so much throughout my childhood, lying straight up and down. Even if it has been nothing to lie about, I still lie as a safeguard mechanism. And I will give false alibis just because I am so afraid.

Rebelling against their family’s norms in secret and living double lives were often motivated by the desire to take part in the same activities as their classmates. To be secretly rebellious implied meeting boys, smoking cigarettes, drinking alcohol, or skipping school. Some of the women went to clubs, festivals, and rave parties in secret and a few dated men they chatted with on the Internet.

Women were sometimes honest with their parents, telling them about important events in their lives such as meeting a boyfriend or having stable relations with young men without any protests from their relatives and families.

#### Spring board to freedom

The women had different strategies to become emancipated by planning their future life, limiting or excluding all contact with their family or relatives, trying to buy themselves time. Some women thought about marriage as a step to freedom, a second hand solution that reduced family control, whereas others moved to their own apartment when they were rather young. This was described as a liberalization process—i.e., getting an education, networking, and saving money. Some of the women studied hard to choose the university of their liking, sometimes far away from their family, giving them periodic freedom during the semesters. Jasmine described her situation:

It is great. Everything is great. Yes, but I wake up, go to school, work out, come home, study a little. I can go out; I can come home again. I can walk down the street without someone watching me. I have failed every exam in this program, but it is great because I am free.

### A continuum of severe consequences

#### Alienation and loneliness

A couple of the women were content with their previous and current living conditions, but for the rest of the women there was a continuum of narratives mirroring how the women struggled with adjustment and uproar over the years which were perceived to infringe their physiological, emotional, sexual, and physical health. Many women described feelings of powerlessness, loneliness, isolation, and no one to turn to. Women described how they were in a vulnerable position, out in the cold and without support from their family. One woman was unable to seek help because she experienced language barriers, whereas other women had trouble trusting social counsellors in school, fearing the consequences if the family would find out. Furthermore, the women reported being ostracized by their family when making their own choices. Shadia clarified:

The thing is that when I or others choose to go our own way, it has consequences. We will be Zero set. Like, everything […] you lose everything. There is nothing.

#### Violence against women

Fear was a common feeling connected to unhealthy family relations or to a violent husband or boyfriend. Many of the women described being terrified of being caught breaking the family’s rules, not knowing what the family or husband were capable of or even fearing for their own lives. Women reported a forewarning of danger and the need to stay one step ahead of a hazardous situation. Threats such as being sent away to their family’s country of origin, violence, and even death threats were described as a motivator to keep the women obeying. Elektra experienced constant threats of violence:

And because I grew up in a Muslim family and always been, like, brainwashed by the punishment of God and sin. Eh. Haram, it’s like sin and disgraceful to have sexual relationships before marriage. It damaged my … sexual identity very much. And it stopped me from, even when I fled from home I received death threats and my father went after me when he heard that I had a boyfriend.

The women told that they were violated and traumatized by their father, brother, or husband and the violence tended to escalate, sometimes leading to attempted murder. Elektra shared her experience:

Eh, ah, what happened was that my son had seen very much violence and what made me move out was that the last time. […] The last time his father choked me like that was in front of him, and I hear my son screaming, it was one of those death. […] It was not a normal scream and he sees that I, like, am passing out and I fell down. I wake up and my son is sitting and wiping me and such. And I think that I am the one that should fucking comfort him; he should not comfort me. I am the adult here.

A majority of the women told that they had experienced severe forms of violence, but not all of them wanted to press charges against their family, boyfriend, or husband. The reasons for this varied; some of them wanted to protect the family from bad rumours and did not dare to call the police, whereas Evin feared exclusion and violent consequences if she reported:

I, like, type … do not want any more contact with him. I do not want to do that reporting thing. I just want to break up with him.

In specific situations, the only choice was to flee; however, the death threats did not always end when they broke with the perpetrators and some of them continued to live with a constant threat of violence from a family member. Three of the women had a protected identity as they were afraid of being found. One woman claimed that she was forced to have contact with her mentally and physically abusive ex-husband because of shared custody.

#### A high price to pay

The women described a variety of negative emotions connected with their life situation, such as deteriorating mental health problems resulting in a reduced quality of life. Feelings of hopelessness and frustration led to depression. Women described an overwhelming feeling of being tired, because they were carrying a heavy load for a long time, leaving them emotionally and mentally burnt-out. They had to pay a high price for their struggle for freedom. The process included building a whole new life, affected by trauma and oppression, and this was perceived as a time-consuming process according to Sara:

It has been a long way back; it is just the last few years that I have started to feel free from my own oppressive thoughts – the oppression that I have been subjected to. And psychologically it has been a very traumatizing upbringing when it comes to control.

One woman felt socially damaged and another one attended psychotherapy to process the trauma she had been exposed to. Several women thought that they were overwhelmed with bad conscience because they resisted their family values and did not fulfill the expectations of becoming an honourable woman.

Most of the women described the process of emancipation as demanding. A few of the women described a deteriorating mental health and two of them tried to commit suicide. The women experienced suicide as the only solution in a hopeless family constellation. Jasmine asserted:

But I, like, did not have the energy. It was just dark. I will not even manage to finish high school. I will never get those grades. I will never be able to move away from here. I do not give a damn anymore; either you kill me or I will do it myself because I do not want to live like this.

## Discussion

A thread through our result is the core concept “Honorable women in becoming”, reflects the many informants live up to a conventional femininity dictated by a patriarchal system, where a woman is expected to be submissive. Beauvoir argues that the male view sees women as feeble, shallow, passive, and docile, and to be independent reduces her femininity and her possibility to please (De Beauvoir, 1949/2012, p. 267). We interpret that our informants act “honorably” by resisting restricting norms, choosing to live autonomous lives, a choice that often endangers their lives and safety. These actions are precisely in line with what de Beauvoir names emancipation. Women’s situation and oppression historically and presently must be placed in a societal context. In addition, women can deliberate themselves if they get the necessary preconditions in terms of making a living and economic independence (Lundgren- Gothlin, 1991, pp. 116–124). In terms of preconditions, in Sweden the political discourse, legislation, and policies are moving forward concerning equal rights for women and men, which allows us to speculate that the means are there, but the policy machinery might not reach all women in Sweden due to power dimensions and axis of inequality such as “race”, sexualities, class, language, ability etc.

In our study, many women described different degrees of suffering such as powerlessness, loneliness, isolation, and alienation. Moreover, fatigue and hopelessness leading to depression occurred in the narratives as well as physical and psychological violence that often escalated over time, making their situation very challenging. In our sample, a couple of the women had considered suicide, one woman fled from home, two women lived in women’s shelter, and one woman had protected identity because of death threats from men in their family, all examples of the severity of violence, and the institutionalized misogyny.

Our findings point to the fact that when young women are perceived to become too Western/European/Swedish, this rebellious act may provoke their families, especially the men in their families, to control them (Grzyb, 2016). Some studies suggest that it is common for these actions to be sanctioned collectively and in some cases be socially as well as legally acceptable (Awwad, 2001). Notwithstanding, the informants in our study realized that the legislation in Sweden gave them the freedom to marry whomever they chose and the right to be engaged in romantic love and to educate themselves. Hence, these autonomous life choices may often collide with their parent’s perceived hierarchical, patriarchal family order and this will shape conflicts (Grzyb, 2016), and may reduce young women’s scope of action and increase their vulnerability (Björktomta, 2019). To decrease the risk for collision between family members, some of our informants adjusted to the family values, but others revolted in secret as to not bring shame to their family in an attempt to present themselves as a respectable woman available for marriage. In families, where it is important to choose a spouse from the clan, community or relatives arrange the marriage; young women who refuse to conform to these norms and instead live their lives autonomously will in some cases be extremely penalized and violated. Although one informant had not experienced serious conflict or control, our findings point at how they, to varying degree, suffered from various consequences that they perceived affected their health.

From our point of view, many of the informants have paid a very high price whether they stayed in their families or broke the contact with family members, a dilemma that is not easily solved. To start with, young women are seldom motivated to break with their family or cultural ties because of the risk of becoming completely isolated, cut off from society, and alone with their problems (Cinthio, 2015; Douki et al., 2003; Juth et al., 2013). Furthermore, various forms of punishment and control over young women’s behaviour become a complex family affair where latent death threats are mixed with love and affection, making it difficult for women to distance themselves and resist their family’s will (De los Reyes, 2003). According to Yakin Erturk (United Nations Human Rights Council, 2007), this collective element makes it extremely difficult to separate the victim from actual or potential perpetrators unless she is willing to break off all relations with her family and begin a new life outside her social frame of reference. A Swedish report about honour violence also points to the collective involvements of families; this report distinguishes between honour violence and other forms of violence against women, which often involves an individual perpetrator (The National Centre for Knowledge on Men’s Violence Against Women, 2011).

A structural dilemma is that honour violence has often been largely overlooked both socially and politically mainly because of its “traditional” or “cultural” dimension and thus shielded by tolerance and respect for multiculturalism (Fildis, 2013). The risk of accusations of racism or cultural stereotyping has often been used to excuse the discriminatory and inhumane practices against women that violate women’s human rights (Fildis, 2013). According to Sen (2005) the state is complicit in crimes of honour and practices of violence against women making state racism against women real. This is in line with Grans (2016) idea that the state might have the obligation to interfere when a person is at risk for or subjected to honour violence; however, from the juridical perspective, honour is a complex issue and interference cannot be done automatically. Grans notes the constraints of autonomous decisions—i.e., hindering the development of relationships with other human beings may violate the right to a private life. The key issue in the right to private life is to establish relations of different kinds, for example, the right to marry or to divorce.

Honour violence is part of a broad spectrum of oppression against women, as a particularity on a continuum of violence against women taking place in all societies (Sen, 2005) and this violence often starts with the men in their family who disapprove of women’s normal life choices. As Cooney (2014) puts it, deciding how to dress and who to marry are human rights for women. Cooney (2014) notes that because men are perceived as superior and women as inferior, this type of violence is associated with the core family and when they are married they belong to their husband. De Beauvoir (1949/2012) argued that men define women not as free agents but according to their relationship with men. Women are not perceived as independent as she is only what the man decides she is. de Beauvoir pointed at the key issue, the dividing line between human freedom and independence and human’s capability to act are limited. Hence, as long as women’s and men’s situations are not equal, freedom is seldom possible (Lundgren- Gothlin, 1991, p.226). The oppressors might camouflage the oppression by making it look like a “natural situation”—i.e., to become honourable women, for de Beauvoir the recipe is to raise consciousness among oppressed groups and to revolt. Humans can also refrain from resistance, sometimes because the common goods (i.e., means) are not there or the oppressed do not or cannot use these means properly for various reasons (Lundgren- Gothlin, 1991, pp. 228–29). For de Beauvoir this implies that freedom is not absolute, and humans’ (women’s) situations and possibilities are restricted both historically and socially, and women were/are constructed as the absolute Object and the absolute Other, which distinguishes the *common* situation for many women (p. 249). During the data collection, we analysed how concrete cases in a given social context informed us what this could mean for women when it comes to values, norms, and demands.

One major dilemma for western feminists concerns how to define so called honour violence without falling into the trap of either culturalising or universalizing the phenomenon (Carbin, 2014). This view acknowledges that honour violence can both be seen as a particular violence against women from ethnic minority communities, or as part of the general violence against women existing in every society (Carbin, 2014; Lund Liebmann, 2020). This wider definition acknowledges the fact that men from Western cultures are as patriarchal and violent as men from “other cultures” or constructing “the backwards other East” as responsible (Çoymak, 2020). Eldén (2003) claims that to portray honour cultures as a specific to Muslim or Arab culture is problematic; the issue of honour violence should be placed in a global framework of violence against women, implying that all violence against women is the same in all patriarchal cultures globally. The Canadian media coverage of honour killings may present a skewed view, making honour a problem for only immigrants, where the “real” problem, that is the patriarchal idea that men can and even have the right to control their girlfriends, wives, or sisters occur in most cultures (Shier & Shor, 2016).

Susan Moller Okin (1998) has critiqued policy approaches based on multiculturalism because it has the power to undermine gender equality and lacks compatibility with feminism and the rights of women. She argued that women should not be disadvantaged because they are women and must be provided the opportunity to live as freely as men. From Moller Okin (1998) point of view, the defence of “cultural collective practices” is likely to have much more impact on the lives of women and girls than on those of men and boys. Moller Okin (1998) wondered why, on liberal premises and within a liberal society, should a cultural group be “entitled to try to live by their ways” if these ways violate the individual rights of their members. From that point of departure, multiculturalism may have the effect of reinforcing gender inequality as there are more powerful group members who can oppress more vulnerable members within the group, such as women (or sexual minorities etc) (Song, 2005). In a recent publication by Hülya Simga (2019), the author claims that patriarchy flourishes on the cultural norms of honour that favours men at the expense of women’s freedom and rights, and from her point of view, human rights are not gender-neutral as women’s rights are neither respected nor elicited in the documents. In line with de Beauvoir’s concept of eternal femininity, a determinate myth, is sustained by a patriarchal society to justify the oppression of women (Lundgren- Gothlin, 1991, pp. 250–51). How women are defined, what myths circulate, depends on the society and the individual’s needs. For example, a woman who transgresses/transcends the ideal of the femininity myth instead of maintaining the immanence fulfilment stays in place in the self-fulfilment myth and risks being seen as unfeminine. From that starting point, our core concept, honourable women in becoming is exact in line with de Beauvoir’s elaborations of the social constructions of women in *The Second Sex*. Our interpretative model concerning how the informants construct their lives and identities and “become women” in relation to the honour culture in the family and the Swedish majority society can be viewed as their way of emancipation in their specific situation, but not the only way. Hence, we believe gender equality norms are not about expressing the supremacy of the west and the backwardness of “others” but to make sure that women’s rights and needs are considered.

Further research, with inclusion of older age groups of women, women with lower educational levels and research on men’s views can broaden the perspectives and understanding on honour.

### Methodological considerations

#### Strengths and limitations

Consistent with Charmaz (2006, pp. 182–183), we endorsed the process of rigour based on credibility, originality, resonance, and usefulness. Credibility and originality went hand in hand and enhanced the quality. The interviews contained rich data, while the uniqueness of adding de Beauvoir’s theory contributed to new insights useful at both individual and structural level. In this study we provide theoretical, methodological, and substantive contributions concerning how the informants’ situations formed their lives. The recruitment was time-consuming, and approximately ten women refrained from being interviewed; the most common reason for refusing was fear of being identified. Key persons at women’s organizations working against violence against women selected and recruited informants *they* thought would be ultimate for the study, but to compensate for the risk for skewed sample, recruitment was expanded. On the other hand, there were probably women who were experiencing more violence or a higher level of control and thus did not want to take part in an interview (Lincoln & Guba, 1985). Hence, the spread across various countries, ages, and social backgrounds increased the width and depth of variation. We recruited a sample of women with various views of restrictions, reprisals and violence. Fourteen informants is a fairly small number and the findings must be interpreted with caution. However, the emergent categories were merged into a core concept as a whole, and individual interviews should not be interpreted in isolation. According to Charmaz (2006), the theoretical saturation is reached when the properties and dimensions within the categories are fully developed and further analysis adds little to the result. During the analysis, we sorted and resorted the data and continued with the codes that were meaningful for our purpose, and we carefully explained the process of the analysis to make the result trustworthy. Saturation may be difficult to reach in most research projects due to time limits and costs (personal and financial) and in every new interview something different will turn up.

In line with Strauss and Corbin (1990, p. 189), all persons in the research team have been present in the analytic sessions where data (such as interviews and memos) were brought back and shared, in the group. Furthermore, the team have partaking in the making of categories and concept. The categories and subcategories are linked together and integrated in the model (Figure 1), and ”it is this tight linkages, in terms of the paradigm features and density of the categories, that give a theory its explanatory power” Strauss and Corbin (1990, p 255). Hence, the emergent core category and the flow chart of the web of actions is, from our point of view, theoretically supported.

There are various ways to interpret transcribed interviews depending on the researchers’ point of view. According to constructivist grounded theory, methodological self- consciousness is part of reflexivity and means that we as the researchers were “inspecting ourselves” during the research process (Charmaz, 2017). For instance, we were aware of our taken—for-granted privileged positions and the risk for othering and we were carefully rethinking how we formulated ourselves during the process, and we perceived the informants as agentic actors to diminish the risk for marginality. Our background as Swedish women, midwives and gender researchers could have influenced how some informants portrayed themselves as more or less vulnerable, by over- or understating their narratives, risks contributing to a polarized image of the emancipated white western woman versus the feminine other, a victim of patriarchal control (Sanberg & Janssen, 2018).

To stimulate theoretical sensitivity, the use of philosophical literature, in this case Simone de Beauvoir’s theory provided ways to approach and interpret the data which is a strategy to increase validation (Strauss and Corbin 1990, pp. 50–51). We believe that Beauvoir’s framework strengthened the complexity of the analysis and the emerging core concept, but Beauvoir’s theories have been criticized for being outdated and not applicable in contemporary society (Bryson, 2003). However, our perspective is that Beauvoir’s theory about women as “the Other” is relevant and sustainable in contemporary society, for instance, by studying the variety of women’s lived experiences, their situations and how individual woman encounters, internalizes, or rejects dominant gender norms (Moi, 2011), as seen among our informants in their ongoing process of forming their own life choices.

## Conclusion

In this small-scale study we have explored how a group of young women at a particular time and place construct their views and actions concerning how they comply with or resist patriarchal chastity norms and control, and how they perceive that these norms affect their lives. Gender constructions are highly present in the narratives, where the emergent core concept, “honorable” women in becoming addresses how the web of collective and individual actions shape “ideal” women. The core of honour (although a complex, confusing and sensitive concept) can be grounded in the interest of a collective unit, in this case a family, which collides with the autonomy of the individual, where the right to privacy fails to protect women from abusive men (Kymlicka, 2002, p. 396). Moreover, this view has excused unwanted isolation, forced modesty, in women who desire to take part in public life. The traditional gender roles in the family, where many women are treated as inferior, might conflict with the equality policies in Sweden (Gender and society https://sweden.se/society/gender-equality-in-sweden/). Hence, discrimination against and control of the freedom of women is practiced, more or less, by virtually all cultures, past and present Song (2005). If the rights of the family are not harming the individual *woman*, it is one way to think and act in line with the emancipation of women.

We have underlined that it is difficult to show the exact breadth of the specific problems related to honour violence. The nature and sensitivity of the topic and the risk for violence and punishment contribute to its invisibility. Despite equality promotion and gender equality ideology in Sweden, our concern is that the authorities might over exaggerate or not act as long as it is seen as the immigrants’, or “the feminine others” problem. The fact that this particular group of women in our study had their roots in the Middle East and East Africa does not mean that men in these cultures are more oppressive towards women compared with men in western culture, but we need to make a nuanced and socially situated understanding of violence against women and seriously listen to women who are describing their reality as restricted without downplaying their testimonies.
